# Trefoil Factor Family Member 2 Expression as an Indicator of the Severity of the High-Fat Diet-Induced Obesity

**DOI:** 10.3390/genes12101505

**Published:** 2021-09-26

**Authors:** Abdelaziz Ghanemi, Mayumi Yoshioka, Jonny St-Amand

**Affiliations:** 1 Functional Genomics Laboratory, Endocrinology and Nephrology Axis, CHU de Québec-Université Laval Research Center, 2705 Boul. Laurier, Québec, QC G1V 4G2, Canada; Abdelaziz.Ghanemi@crchudequebec.ulaval.ca (A.G.); mayumi.yoshioka@crchudequebec.ulaval.ca (M.Y.); 2 Department of Molecular Medicine, Faculty of Medicine, Laval University, Québec, QC G1V 0A6, Canada

**Keywords:** Trefoil Factor Family Member 2, expression, indicator, high-fat, diet, obesity

## Abstract

Trefoil Factor Family Member 2 (TFF2) belongs to TFF family peptides that includes TFF1, TFF2, TFF3. TFF2 is mainly known for its roles in the mucosal protection. In the context of obesity and high fat diet (HFD), *Tff2* has been characterized as a HFD-induced gene. The knock-out of *Tff2* in mice lead to the protection from HFD-induced obesity with a metabolic profile towards a negative energy balance. Such HFD-specific expression gives *Tff2* a pattern worth exploring in biomedical research. Indeed, measuring TFF2/*TFF2*/*Tff2* expression in biological samples following the ingestion of high-fat diet reflects the biological “responsiveness” to the lipids ingestion and would reflect the severity of obesity establishment afterwards. Such property could be explored for instance to screen animal models, evaluate the predisposition to HFD-induced obesity as well as in biomedical and clinical applications. Results might advance obesity research especially in terms of understanding lipid-induced signals, appetite control and adiposity storage.

Obesity represents a growing challenge for health professionals and officials which represents a risk factor for a variety of diseases (including during the ongoing COVID-19 crisis [1,2,3,4]) and various diseases [5,6,7,8]; and it is also considered a disease itself [9]. It also represents a huge economic burden [10,11]. The main challenging pattern facing the development of obesity research and therapies is the limited understanding of its molecular and cellular pathways [12,13]. Therefore, providing new molecular tools to explore obesity, its development and its pathogenesis remains of a high importance. Within this piece of writing, we describe a potential application of trefoil factor family member 2 (TFF2/*Tff2*) expression pattern related to high fat (HF) diet (HFD). 

TFF2 belongs to TFF family peptides that includes TFF1, TFF2 and TFF3 [14,15]. TFF2 is mainly known for its roles in the mucosal protection including in the gastrointestinal tracts [15,16,17], but it is also implicated in a variety of functions including anti-inflammatory process [18], tissue repair [19] and cancer [20]. Interestingly, recent studies have highlighted metabolic implications of TFF2 especially in the context of obesity and HFD. Indeed, using functional genomics approaches [21], *Tff2* has been characterized as a HF-induced gene in mice intestinal mucosa. The HF specificity has been revealed through an experimental design that used fasted status (instead of low-fat) as a control to which both HF and low-fat fed mice have been compared [22,23]. Indeed, the gene expression, studied based on serial analysis of gene expression and confirmed with microarray analysis, revealed that in the intestinal mucosa the *Tff2* is overexpressed following the ingestion of a HF meal and not a low fat meal [22,23]. Therefore, highlights *Tff2* as a HF specifically-induced gene.

In order to elucidate the implications of TFF2 in the context of obesity, and more specifically in the HF-diet obesity, *Tff2* knock-out (KO) mice were challenged with HFD [24]. The study has shown that the *Tff2* KO mice, compared to the wild-type (WT) mice, are in fact protected from HFD-induced obesity with an increased lipids excretion as well [24] which correlates with the exacerbation of weight loss by TFF2 deficiency shown by Judd et al. [25]. Moreover, the metabolic exploration of key metabolic tissues of these mice revealed mechanisms that explain such protection. Indeed, *Tff2* KO mice have a metabolic phenotype towards an increased energy expenditure with reduced energy storage [26]. In our recent review [27], we have detailed a hypothesis that aims to explain how HFD induces *Tff2* overexpression and at the same time the KO of this same gene, *Tff2,* lead to the protection from the HF-diet-induced obesity via metabolic changes. Briefly, the TFF2 expression would be a signal leading to metabolic adaptation, which facilitates the lipid digestion, anabolism and storage. Therefore, HFD would induces its overexpression to facilitate the digestion and the anabolism of lipids coming from such HFD, whereas *Tff2* KO would deprive the metabolic machinery from molecular tools required to use the ingested lipids through an increased lipid absorption and storage, which leads to a protection from the HFD-induced obesity. It is worth pointing that gut microbiota, which contributes significantly to metabolic disorders [28] including obesity [29], impaired glucose [30] and lipid metabolism [31], can also be altered by diets [32,33] including HFD [34,35]. In the obesity context, the interactions between TFF2 and gut microbiota [36] could be involved in the mechanisms of HFD-induced obesity. Therefore, TFF2 would represent a molecular mechanistic link between HFD and obesity development [27]. 

Based on such properties of *Tff2* induction by HDF and its implications in HFD-induced obesity, potential applications can derive from and range from biomedical research to clinical practice (Figure 1). The concept would be to challenge biological systems (animals, cell cultures, isolated tissues, etc.) with HFD followed by the measure of *Tff2* or TFF2 expression in the intestinal mucosa [22,23], blood [37] or other tissues [38,39,40]. This could allow for instance to evaluate the “predisposition” to develop HFD-induced obesity based on the expression intensity of TFF2/*Tff2* following HFD. Obesity animal models are diverse [41], among them different species have been used to generate animal models of HFD-induced obesity. Within this context, measuring *Tff2* expression following the ingestion of a HFD could represent a standard approach to compare the different animal models and therefore optimize the selections of the one(s) suitable to build the obesity model for the experiments depending on the experimental contexts and goals. The same principle can be applied to select, following a genetic modifications (KO, overexpression, etc.), the animals to be used for breeding and used for the obesity-related studies. 

For clinical perspectives, we can estimate the risk of HFD-induced obesity in individuals by the same approaches. For the pharmacological studies and research applications, obesity drugs can be tested as a purpose to reduce *Tff2* expression and therefore mimic *Tff2* KO and lead to a metabolic profile similar to the one seen in *Tff2* KO mice described above (protection for HFD-induced obesity) [24,26]. Therefore, adapt the individuals’ diet, not only in terms of lipids content but even depending of lipids types. Indeed, for the pharmacological studies and research applications such measures can be used also to adapt a diet, test a drug or evaluate a treatment in the context of HFD-induced obesity. This might be achieved by measuring TFF2/*Tff2* expression depending on the type of lipids that can also be tested in cells to study the molecular changes. Therefore, the studies would go deeper by comparing, among the HFD, the different types of lipids and test a variety of combination to gain new knowledge on links between diets composition and its ability to induce obesity (through *Tff2* expression) especially while comparing diets that have similar number of calories. Within this context, the variety of functional genomics methods [42,43] allowing to measure the *Tff2* expression are molecular tools that provide a flexibility to such applications as well. 

We believe that the measure of TFF2/*TFF2*/*Tff2* expression level in response to HFD could expand our cellular and molecular understanding of obesity and strengthen therapeutic research especially that TFF2 could be a lipid-specifically induced signal we are yet to confirm to complete the puzzle of fat-induced signals, appetite control and adiposity storage; all key elements in energy homeostasis and obesity development. 

## Figures and Tables

**Figure 1:**
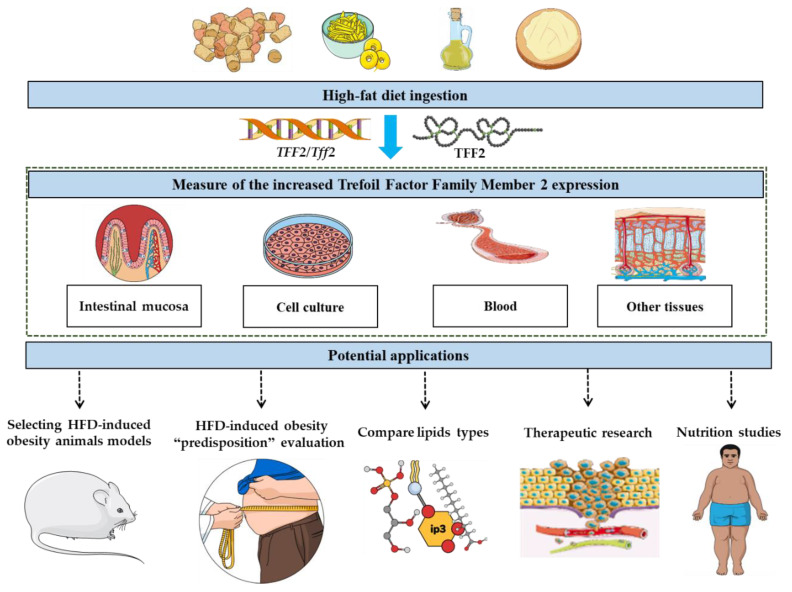
Measuring Trefoil Factor Family Member 2 (TFF2/*TFF2*/*Tff2*) expression in biological samples following the ingestion of high-fat diet reflects the biological “responsiveness” to the lipids ingestion and would reflect the severity of obesity establishment. Such property could be explored for instance to screen animal models, evaluate the predisposition to high-fat diet-induced obesity as well as in biomedical and clinical applications.

## Data Availability

Not applicable.
